# The Influence of Paid Memberships on Physician Rating Websites With the Example of the German Portal Jameda: Descriptive Cross-sectional Study

**DOI:** 10.2196/39259

**Published:** 2023-04-04

**Authors:** Friedrich Aaron David Armbruster, Dörthe Brüggmann, David Alexander Groneberg, Michael Bendels

**Affiliations:** 1 Institute of Occupational, Social and Environmental Medicine Goethe University Frankfurt Germany

**Keywords:** physician rating websites, physician rating portals, paid influence, Germany

## Abstract

**Background:**

The majority of Germans see a deficit in information availability for choosing a physician. An increasing number of people use physician rating websites and decide upon the information provided. In Germany, the most popular physician rating website is Jameda.de, which offers monthly paid membership plans. The platform operator states that paid memberships have no influence on the rating indicators or list placement.

**Objective:**

The goal of this study was to investigate whether a physician’s membership status might be related to his or her quantitative evaluation factors and to possibly quantify these effects.

**Methods:**

Physician profiles were retrieved through the search mask on Jameda.de website. Physicians from 8 disciplines in Germany’s 12 most populous cities were specified as search criteria. Data Analysis and visualization were done with Matlab. Significance testing was conducted using a single factor ANOVA test followed by a multiple comparison test (Tukey Test). For analysis, the profiles were grouped according to member status (nonpaying, Gold, and Platinum) and analyzed according to the target variables—physician rating score, individual patient’s ratings, number of evaluations, recommendation quota, number of colleague recommendations, and profile views.

**Results:**

A total of 21,837 nonpaying profiles, 2904 Gold, and 808 Platinum member profiles were acquired. Statistically significant differences were found between paying (Gold and Platinum) and nonpaying profiles in all parameters we examined. The distribution of patient reviews differed also by membership status. Paying profiles had more ratings, a better overall physician rating, a higher recommendation quota, and more colleague recommendations, and they were visited more frequently than nonpaying physicians’ profiles. Statistically significant differences were found in most evaluation parameters within the paid membership packages in the sample analyzed.

**Conclusions:**

Paid physician profiles could be interpreted to be optimized for decision-making criteria of potential patients. With our data, it is not possible to draw any conclusions of mechanisms that alter physicians’ ratings. Further research is needed to investigate the causes for the observed effects.

## Introduction

According to a representative survey commissioned by the Bertelsmann Foundation in Germany, more than 90% of respondents see a deficit in information availability on choosing a physician [1]. At the same time, more than half the population assumes strong differences in the quality of care among different physicians [2]. With a patient-generated rating system, around 25 physician rating websites in Germany are attempting to provide a platform for choosing a physician [3].

The influence of these rating portals on choosing a physician is significant; a survey by the Friedrich Alexander University of Erlangen Nuremberg indicated that 65.5% of physician rating portal users made their choice of physician on the basis of the information provided [4]. Thereby, the selection of a physician seems to be highly subjective. In a study by Carbonell and Brand [5], it was shown that comments and ratings from other users were more influential than facts, such as specialization or experience.

Another study investigated the influence of ratings more specifically. In a simulation of a physician rating platform, the decision-making behavior of study participants was examined before and after they were presented with ratings from fictitious physicians. Participants were shown to be significantly more likely to choose profiles with many recommendations and ratings; negative ratings as well as low number of ratings had a deterrent effect on the participants [6].

Consequently, the reputation on physician rating websites also gains an economic aspect [7]. In the United States, cases have been reported in which physicians were engaging agencies to improve their own representation on a rating portal [8].

In Germany, Jameda.de is the most popular and well-known physician rating portal, with around 6 million monthly users and approximately 2 million patient ratings [9,10]. With 87.2%, Jameda lists the majority of physicians practicing in outpatient care [9]. Listed physicians obtain a profile on which patients can leave ratings in German school grades, ranging from 1 (best) to 6 (worst). Each patient’s rating includes at least some text and grades in 5 mandatory categories, from the mean values of which the overall grade of an individual rating is calculated. Ratings in a further 12 categories can be given voluntarily, and the physician can be recommended to other patients. The average value of these individual reviews is used to calculate the overall marks of the profiles. The algorithm for determining recommendation quotas or the number of colleague recommendations is not known; users are only presented with a simple numerical value. Ratings older than 4 years are archived and thus excluded from calculations. Reported ratings can either be suspended, deleted, or rereleased after review by Jameda.

However, Jameda’s role as a neutral referral platform is not undisputed. Jameda, like other German review websites, offers monthly paid membership packages (Gold, Gold Pro, and Platinum) for physicians. Each of these packages provided a gradual expansion in functionality. Starting with Gold, it was possible to add a profile picture. Higher paying membership plans also included, for example, appointment allocation services and the option for web-based consultations. Jameda contradicts claims [11] that a paid membership plan has a positive effect on ratings or list rankings [12].

So far, the influence of paid memberships has not been the focus of scientific investigation. The goal of this study was to investigate whether a physician’s membership status has an impact on his or her quantitative evaluation factors and to possibly quantify these effects. Specifically, the key parameters—overall grade, grade distribution, number of evaluations, recommendation quota, colleague recommendations, and profile views—were analyzed as a function of membership status (ie, nonpaying, Gold, and Platinum).

## Methods

### Data Acquisition

Between January 31, 2020, and February 2, 2020, a total of 25,549 Jameda physician profiles were retrieved via the provided search mask on the jameda.de website. Regions and medical disciplines were selected to result as many paying members as possible for the smallest amount of search queries.

Specifically, profiles with an overall score from the following 12 most populous cities in Germany were acquired: Berlin (n=5456, 21.4%), Hamburg (n=3526, 13.8%), Munich (n=3057, 12%), Cologne (n=2171, 8.5%), Frankfurt (n=1516, 5.9%), Stuttgart (n=1182, 4.6%), Düsseldorf (n=1481, 5.8%), Leipzig (n=1058, 4.1%), Dortmund (n=793, 3.1%), Essen (n=971, 3.8%), Bremen (n=986, 3.9%), and Dresden (n=1022, 4%).

In addition, the query delivered 2330 (9.1%) profiles from the surrounding urban regions.

Due to technical restrictions on the part of the provider, only a maximum of 90 profiles could be read out for each defined search term. To increase the sample size, the search term was specified by the respective city districts.

In terms of content, these 8 disciplines were selected: “internal medicine and general medicine” (n=8032, 31.4%), dentistry (n=7744, 30.3%), gynecology (n=2519, 9.9%), orthopedics (n=2068, 8.1%), ophthalmology (n=1391, 5.4%), dermatology (n=1375, 5.4%), neurology (n=1063, 4.2%), and plastic or aesthetic surgery (n=385, 1.5%). The searches yielded 972 (3.8%) profiles that were primarily assigned to other specialties. These profiles were also retained. The result sorting was left at default (“relevance”). Profiles that had been acquired more than once could be identified by means of the unique Jameda-specific profile ID, and of these, only the most recent acquisition was included in the evaluation. Location and specialty assignment were extracted from the internet address. The membership status was extracted from the website source code. Since both Gold and Gold Pro were displayed as Gold, it was not possible for us to distinguish between them.

### Data Analysis

For analysis, the profiles were grouped according to member status (nonpaying, Gold, and Platinum) and analyzed according to the target variables—physician rating score, individual patient’s ratings, number of evaluations, recommendation quota, number of colleague recommendations, and profile views. Individual grades were taken from the grade report, and the total number of evaluations were calculated from the sum of the number of these individual ratings.

Significance testing of group-specific means with SDs was performed using a single factor ANOVA test followed by a multiple comparison test (Tukey test). Data analysis and visualization were performed using Matlab (The MathWorks, Inc).

## Results

A total of 21,837 (85.5%) nonpaying profiles, 2904 (11.4%) Gold, and 808 (3.2%) Platinum member profiles provided the data base for our analysis. The proportion of profiles with paid membership plans was thus 14.5% (n=3,712).

### Overall Physician Rating

**Figure 1:**
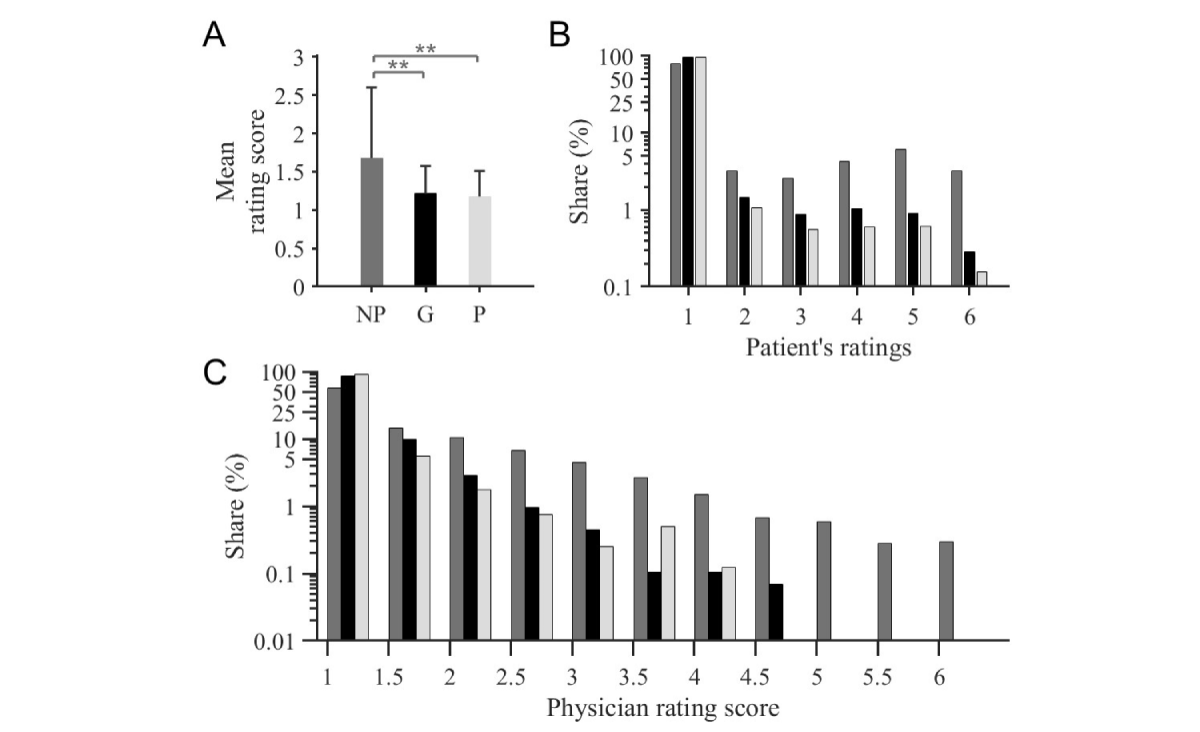
Analysis of physician-specific total and individual scores. (A) Mean physician rating score grouped by membership status. The higher the membership status, the significantly better the mean rating (mean and SD are represented; ANOVA; ** represents *P*<.001). (B) The group-specific distribution of the patient’s rating in semilogarithmic representation similarly documents a relative overrepresentation of grade 1 ratings among paying members. (C) The group-specific distribution of physician rating score in semilogarithmic representation documents an accentuated relative overrepresentation of grade 1 among paying members. The higher a member’s status (Platinum > Gold), the more pronounced this effect becomes. G: Gold; NP: nonpaying; P: Platinum.

The mean overall physician grade was 1.68 (SD 0.92) for nonpaying profiles, 1.21 (SD 0.36) for Gold members, and 1.18 (SD 0.33) for Platinum members; the group-specific mean scores between nonpaying profiles and paid members differed highly significantly (ANOVA; *P*<.001). No statistical significance was found for the mean overall physician score in between Gold and Platinum members (Figure 1A).

The group-specific distribution of physician rating score (N=25,549) documented an accentuated relative overrepresentation of grade 1 in paid members (Gold: 2489/2904, 85.7% and Platinum: 736/808, 91.1%) compared to nonmembers (12604/21837, 57.7%) and a relative underrepresentation of the remaining grades in comparison with nonmembers (Figure 1C). The higher a member’s status, the more pronounced this effect was across the entire grading scale: 3.3%( 725/21837) of nonpaying profiles and only 0.2% (5/2904) and 0.1%( 1/808) of Gold and Platinum members, respectively, had an overall grade of 4 or worse. In our sample, no Platinum profile had a total grade of 4.5 or worse.

### Distribution of Patient’s Reviews

**Figure 2:**
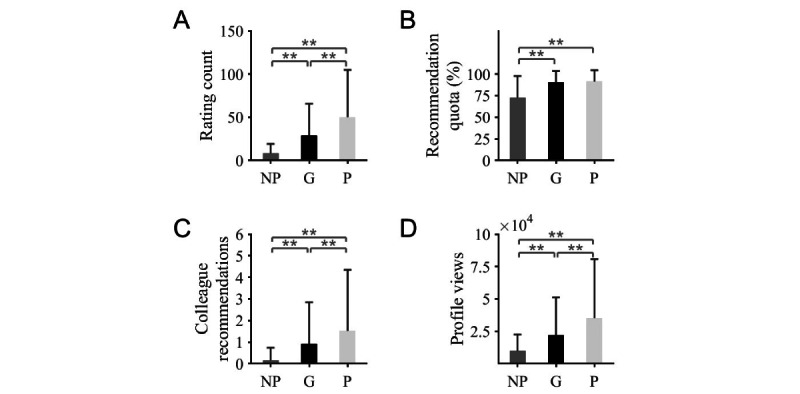
Analysis of nongrade criteria. The higher the membership status, the significantly higher the number of ratings. (A) Recommendation quota; (B) Colleague recommendations; (C) Number of profile views; (D) Mean and SD values for profile views; ANOVA; ** represents *P*<.001. A significant difference was found in between the paid memberships, but no significant different was found for the recommendation quota. G: Gold; NP: nonpaying; P: Platinum.

There were 299,579 patient ratings distributed among 174,730 (58.3%) nonpaying members, 84,319 (28.1%) Gold members, and 40,530 (13.5%) Platinum members. The mean number of ratings per physician was 8.0 (SD 11.2) for nonpaying profiles, 29.0 (SD 36.7) for Gold members, and 50.2 (SD 54.6) for Platinum members; group-specific means differed highly significantly (ANOVA; *P*<.001; Figure 2A).

The group-specific distribution of patient reviews documented a relative overrepresentation of grade 1 reviews among paying members (Gold: 80,482/84,319, 95.4% and Platinum: 39,319/40,530, 97%) compared to 80.5% (140,695/174,730) of the nonpaying members and a relative underrepresentation of the remaining graded reviews compared with nonmembers (Figure 1B). The underrepresentation accentuated with worse patient reviews and higher membership status. The proportion of individual scores of 4 or lower was 13.7% (23,975/174,730) in the nonpaying group and 2.2% (1885/84,319) and 1.4% (555/40,530) in the Gold and Platinum member groups, respectively.

### Recommendation Quota and Colleague Recommendations

A total of 7228 colleague recommendations were found, which were subdivided into 3326 (46%), 2675 (37%), and 1227 (17%) recommendations for nonpaying members, Gold members, and Platinum members. The mean number of colleague recommendations was 0.15 (SD 0.59) for nonpaying profiles, 0.92 (SD 1.92) for Gold members, and 1.52 (SD 2.83) for Platinum members; the differences in means turned out to be highly significant (ANOVA; *P*<.001; Figure 2C).

There were 21,475 (84.1%) physician profiles with a recommendation quota indicated; the range of values was between 0% and 100%. The mean recommendation quota was 72.5% (SD 25.2%) for nonpaying members and 90% (SD 13.5%) and 91.2% (SD 13.2%) for Gold and Platinum members, respectively (Figure 2B); the group-specific difference in mean values between nonmembers and paid members turned out to be highly significant (ANOVA; *P*<.001; Figure 2B).

### Visit Counts

A total of 306,630,270 profile views subsumed into 214,955,367 (70.1%), 63,271,297 (20.6%), and 28,403,606 (9.3%) views for nonpaying profiles, Gold members, and Platinum members. The range of values was between 45 and 518,691 calls per profile. The mean number of profile views was 0.98x10^4^ (SD 1.3x10^4^) for nonpaying profiles, 2.2x10^4^ (SD 2.9x10^4^) for Gold members, and 3.5x10^4^ (SD 4.5x10^4^) for Platinum members; the differences in mean values turned out to be highly significant between all groups (ANOVA; *P*<.001; Figure 2D).

## Discussion

### Overview

In this analytical, descriptive cross-sectional study, the most important quantitative rating indicators of the Jameda platform were analyzed group-specifically according to nonpaying, Gold, and Platinum membership. For this purpose, a sample of 25,549 profiles were examined. This corresponds to a share of 16.2% among approximately 157,300 physicians practicing in outpatient care in Germany [13].

### Rating Correlates With Membership Status

Statistically significant differences were found between paying (Gold and Platinum) and nonpaying profiles in all parameters we examined.

Except for the recommendation quotas and mean overall ratings, significant differences were also found within the paying profiles in the parameters, number of evaluations, and number of colleague recommendations (Figure 1A, Figure 2A, and Figure 2C).

The higher a physician’s membership status was (nonpaying, Gold member, or Platinum member), the significantly better were the evaluation parameters.

### Implications

The decision-making characteristics mentioned in the introduction [6] seem to apply specially to paid profiles. Overall, paid profiles had significantly more ratings but fewer negative ratings than nonmembers. The recommendation quota was also significantly higher among paying members compared to nonpaying profiles. In addition, Jameda presents presumably valuable “colleague recommendations.” These were mainly dedicated to paid profiles (Figure 2C). The identity of these colleagues is not visible to the user.

### Why Do Paid Profiles Perform Better?

This cannot be clarified beyond doubt with the data available to us, since we only performed a descriptive study. However, there was a conspicuous absence of critical individual reviews for paying profiles (Figure 1C).

Assuming that physicians with a paid package are rated similarly to nonpaying physicians, an active mechanism seems possible. Physician rating websites have the ability to suspend or even delete ratings, thus eliminating them [14]. There are various reasons to initiate a deletion of a rating, but one way toward deletion is a report by the physician. The rating portal then decides on the outcome of the deletion process. From a previous study on another portal, we know that the reviews affected by deletion are mainly negative reviews [15]. This thesis is in line with recurring allegations, which accuse Jameda of deleting critical posts or requiring the submission of written proof of treatment [16]. The exact decision criteria for deletion or suspension are not comprehensible for the user of the portal.

This could occur more frequently with paid profiles, as those physicians may consider their representation on Jameda to be important. For example, it has been observed that a professional social media account correlates with a high number of ratings [17]. Physicians generally attaching less importance to review portals might be less inclined to invest monthly in a paid membership or to take action against negative reviews. Whether nonpaying members are less likely than paying members to report critical reviews is not possible for us to test due to lack of data.

### Why Do Paid Profiles Have More Reviews?

In general, the number of ratings on physician rating portals increased over time [18], but profiles with paid memberships have particularly high numbers of ratings. Here a self-reinforcing effect seems possible.

Since patients look for physicians with many and good ratings [6] on physician rating websites, they might choose paying profiles more often and rate them afterwards.

Another possible explanation is that part of the reviews could also be purchased. On the internet, several websites offer to create reviews for the Jameda portal [19,20]. An assessment of how many reviews have been created by marketing agencies and whether this is specific to paid profiles is not possible due to a lack of data.

The impact of a paid membership seems to be noticeable to the physician: Steinfort [21] concludes in an article in the German journal *Gynecology and Obstetrics* about physician rating portals, “a premium status on physician rating portals guarantees a high inrush of new, but also very flexible and transient patients” [21].

### Business Model

Thus, the business principles of some commercial physician rating websites seem to rely on questionable presumptions by the members. To be represented as favorably as possible, some physicians may think that merely paying a rating portal leads to a better standing. In this respect, on other physician rating websites, paying physicians can also partially hide negative ratings for the patient [22]. This raises the question of the extent to which the data presented to the user on physician rating websites can be trusted.

### Methodological Limitations

A fundamental limitation of the study is that it is purely descriptive. No conclusions can be drawn about any (possibly active) mechanisms for changing rating indicators post acquisition of a premium package. Therefore, the authors do not claim to have identified fraud mechanisms of a platform in this study; they supplied descriptive data to gain more information.

A further limitation of the study design is that the search queries were restricted to the 12 largest cities in Germany. Compared to the results from a study by Emmert and McLennan [18], significantly more reviews were found per profile than what Emmert and McLennan found in 2019, which might indicate a sampling bias [18].

Furthermore, we can only separate 3 of the 4 membership plans. It is not possible for us to differentiate between Gold and Gold Pro. An assessment of the rejected or deleted ratings is not possible due to a lack of data. Finally, we only investigated one physician rating website, so it is unclear whether this is true for other portals or even for non-German physician rating websites.

### Conclusions

Overall, we can conclude that profiles of paid members seemed to be optimized for decision-making characteristics of potential patients in all evaluation parameters analyzed by us. With one exception, these effects increased with increasing pay status. High call rates of Gold and Platinum profiles confirm the increased patient interest. Therefore, the results seemingly contradict Jameda’s claim of being a neutral rating platform (Jameda’s quality promise mentions “We treat all physicians the same,” and “Ratings are not for sale”) [12].

Rather, Jameda fulfills the criteria of an advertising platform for paying physicians. In this context, the nonpaying profiles seem to serve as a contrast to the paying members and are thus necessary for the business model of this platform. Due to the anonymity of the ratings and nontransparency of some other parameters, a well-founded physician counterposition is prevented. More analyses of different physician review websites are needed as a next step toward systematization.
